# Measuring linkage quality with hashed identifiers: An example using PAYE - ASHE

**DOI:** 10.23889/ijpds.v8i2.2236

**Published:** 2023-09-14

**Authors:** Hannah Goode, Jen Hampton, Rachel Huck

**Affiliations:** 1 Great Ormond Street Institute of Child Health, University College London, London, United Kingdom

## Objectives

ADR-UK supplied the Wage and Employment Dynamics (WED) research team with Pay as You Earn (PAYE) data linked to Annual Survey of Hours and Earnings (ASHE) data. This presentation explains the methodology taken to link PAYE and ASHE databases together, along with quality assurance and analysis of the linked data.

## Method

The data were primarily linked using deterministic linkage methods on an encrypted National Insurance Number (NINo) variable. A total of four Pay as You Earn tables were linked to the ASHE data.

Where a PAYE table contained an encrypted NINo variable, the PAYE table was linked to the ASHE database using exact matching on NINo. Where a PAYE table did not contain an encrypted NINo variable, the table was linked to another PAYE table with an existing NINo variable first and then linked to the ASHE data.

## Results

As multiple HMRC tables were linked to the ASHE database, the linkage rate for each table varies. However, at least 0.93% of each PAYE table linked to the ASHE database. As the ASHE data is based on a 1% sample of employee jobs taken from HMRC’s PAYE records, these linkage rates are considered relatively high.

To compensate for loss of variables needed to calculate linkage quality, the data was filtered into categories on the ‘sex’ and ‘age’ variables and the linkage rates analysed. Bias analysis associated to records with a missing NINo value was also investigated. When the records with a missing NINo value were excluded from calculations, the linkage rate for each table rose to 1%.

## Conclusion

This data will be used to assist research outputs that focus on low-pay labour markets and wage progression, wage inequalities and employment. The presentation will also discuss challenges overcome and lessons learnt from this project, with suggestion of how this information can be used to improve ADR-UK projects going forward.

